# Altered Prefrontal and Inferior Parietal Activity During a Stroop Task in Individuals With Problematic Hypersexual Behavior

**DOI:** 10.3389/fpsyt.2018.00460

**Published:** 2018-09-25

**Authors:** Ji-Woo Seok, Jin-Hun Sohn

**Affiliations:** ^1^Department of Counseling Psychology, Honam University, Gwangu, South Korea; ^2^Department of Psychology, Brain Research Institute, Chungnam National University, Daejeon, South Korea

**Keywords:** problematic hypersexual behavior, executive control, Stroop task, functional magnetic resonance imaging, dorsolateral prefrontal cortex, inferior parietal cortex

## Abstract

Accumulating evidence suggests a relationship between problematic hypersexual behavior (PHB) and diminished executive control. Clinical studies have demonstrated that individuals with PHB exhibit high levels of impulsivity; however, relatively little is known regarding the neural mechanisms underlying impaired executive control in PHB. This study investigated the neural correlates of executive control in individuals with PHB and healthy controls using event-related functional magnetic resonance imaging (fMRI). Twenty-three individuals with PHB and 22 healthy control participants underwent fMRI while performing a Stroop task. Response time and error rates were measured as surrogate indicators of executive control. Individuals with PHB exhibited impaired task performance and lower activation in the right dorsolateral prefrontal cortex (DLPFC) and inferior parietal cortex relative to healthy controls during the Stroop task. In addition, blood oxygen level-dependent responses in these areas were negatively associated with PHB severity. The right DLPFC and inferior parietal cortex are associated with higher-order cognitive control and visual attention, respectively. Our findings suggest that individuals with PHB have diminished executive control and impaired functionality in the right DLPFC and inferior parietal cortex, providing a neural basis for PHB.

## Introduction

Problematic hypersexual behavior (PHB) refers to the inability of an individual to control inappropriate or excessive sexual fantasies, urges, or behaviors that cause subjective distress or impairments in daily functioning (1–3). Individuals with PHB can contract sexually transmitted diseases or experience unwanted pregnancies from promiscuous sexual relations (4, 5). PHB typically begins in late adolescence or early adulthood, is thought to be chronic or episodic, and mainly affects men (4). The disorder has an estimated prevalence of 3–6% among the community and college students in the US (6–8). In Korea, about 2% of all college students have PHB (9).

The nosology and optimal diagnostic criteria for PHB remain controversial. Whether PHB can be conceptualized as a behavioral addiction, impulse control disorder, or another psychiatric disorder continues to be a topic of debate (10). Regardless of whether PHB is best described as one of those disorders, it shares similar psychological characteristics (i.e., craving, withdrawal, and loss of control) with other forms of problematic excessive behavior, such as gambling disorder and internet gaming disorder (3, 11–14).

Addictive and compulsive behaviors including gambling disorder and internet gaming disorder have been speculated to be related to a loss of control. Specifically, loss or impairment of executive control is a critical characteristic of problematic excessive behavior. Indeed, previous studies have identified a significant correlation between the two (15, 16). A study on pathological gambling demonstrated that individuals with the disorder performed poorly on the reverse Stroop task (16), suggesting that pathological gambling behavior may be due to impaired executive control, which results in an inability to inhibit irrelevant information during such tasks. Similarly, another study revealed that relative to control participants, individuals with internet gaming disorder exhibited impaired executive control associated with diminished medial frontal activation (15).

Emerging evidence also suggests that executive control impairments occur in PHB (17, 18). One brain imaging study demonstrated that participants with PHB had difficulties with impulse control in a go/no-go task and exhibited a higher degree of mean diffusivity in the superior frontal region (17). In a pilot study, Reid et al. (18) used questionnaire responses to identify a specific relationship between executive control and PHB, observing an association between diminished executive control and PHB; however, contradictory results were obtained in a subsequent study (19) that utilized standardized neuropsychological tests to assess executive control.

Since executive function results among individuals with PHB are inconsistent, additional works need to be conducted to provide conclusive findings. Therefore, our aim was to resolve the aforementioned discrepancies among previous studies by using psychological tests and neuroimaging.

The color-word Stroop test was initially designed to assess executive control ability and has generally been used to identify individuals with brain damage that has affected interference-control processing (20). In the Stroop task, participants are instructed to name the font color of a series of color words, and the response time and error rate are used as outcome measures. Since word reading is a more dominant process than color naming in incongruent conditions (e.g., RED printed in blue font), participants exhibit longer reaction times and higher error rates than in congruent conditions (e.g., RED printed in red ink). Several neuroimaging studies have demonstrated that the Stroop task activates a distributed neural network of brain regions including the prefrontal cortex, parietal lobe, motor areas, and temporal lobe (21–23).

The most consistently supported finding is that the prefrontal cortex plays a key role in Stroop performance (24). This area is involved in executive functions and other higher-order cognitions, which are the main neural correlates of problematic excessive behavior (14). Several researchers have reported that individuals with problematic excessive behavior have anatomical and functional disruptions in the prefrontal cortex. This region is known to be implicated in impulse regulation, so disruptions in this area underlie problematic excessive behavior and account for the erosion of free will (25).

Since the Stroop task requires executive control ability and individuals with PHB have decreased control over their sexual behaviors, we hypothesized that the PHB group would show poorer Stroop task performance compared to the control group. Specifically, these differences would be larger in the incongruent condition. We also predicted that there would be larger differences in brain activations associated with executive control, such as in the prefrontal cortex.

## Materials and methods

### Participants

This study was approved by the Institutional Review Board of Chungnam National University (Approval number: 201309-SB-003-01; Daejeon, S. Korea), and all participants provided written informed consent prior to enrollment. Twenty-three men with PHB (mean age = 26.12, SD = 4.11) and 22 healthy men (mean age = 26.27, SD = 3.39) participated in the functional magnetic resonance imaging (fMRI) experiment. Some of the participants attended in another study, i.e., the sexual craving experiment conducted in our laboratory (26). Roivainen (27) reviewed recent large-scale studies and found gender differences in processing speed and cognitive factors. Specifically, females have advantages in processing speed tests involving alphabets and rapid naming tasks while males are faster with reaction time tasks and finger tapping. Given these known gender disparities, we chose to include a male-only group in our study.

All participants were right-handed, native Korean speakers, and had no past or present major neurological injury or illness as evaluated with a self-report questionnaire. Prior to inclusion in the study, an experienced psychiatrist administered structured psychiatric interviews to all participants using the proposed PHB diagnostic criteria utilized in previous studies (2, 28) and the DSM-5 criteria (Supplementary Materials, Table S1). Individuals with PHB met the proposed PHB diagnostic criteria and were free from any other axis I disorder based on the DSM-5 (29). All PHB participants were not involved in any treatment for their disorder.

Twenty-two healthy controls with similar demographics to those of the subjects were recruited from the community via advertisements and flyers.

The Sexual Addiction Screening Test-R (SAST) (28) and the Hypersexual Behavior Inventory (HBI) (30) were used to examine PHB severity in each participant and to identify any relationship between PHB severity and neural responses to the Stroop interference task. The reliability of the SAST-R and HBI have been previously calculated as Cronbach's α = 0.91 and 0.96, respectively (28, 30). The SAST-R contains 20 questions designed to assess sexual addiction tendencies; total scores range from 0 to 20 points, with higher scores indicating more severe addiction. The HBI comprises 19 questions, and the total score ranges from 19 to 95 points. Reid et al. (30) suggested a total score ≥53 as the cutoff for hypersexual disorders. All PHB participants in this study scored above the cutoff for HBI. Individuals with PHB had an average SAST-R score of 11.3 (SD = 3.3) and an average HBI score of 54.4 (SD = 7.3).

Participant demographic characteristics and sexual activity information for the previous 6 months are presented in Table 1. The PHB group showed significantly earlier age of first sexual intercourse and more number sexual partners, frequent sexual intercourse, masturbation, and viewing pornography per week compared to the control group. Also, PHB group showed significantly higher score on SAST-R and HBI.

**Table 1:** Demographic characteristics.

	**Control group**	**PHB group**	***t*-values or**
	**(*n* = 22)**	**(*n* = 23)**	**chi-square values**
Age (years)	26.3 (3.4)	26.1 (4.1)	
**MARITAL STATUS**^a^			
Single	50.0	47.8	0.30
In a relationship	41.0	43.5	
Engaged/Married	9.0	8.7	
Education (years)	16.3 (3.0)	15.6 (4.1)	0.65
WAIS-R total IQ^b^	108.2 (7.1)	110.3 (7.6)	0.95
Age of first sexual intercourse (years)	20.3 (3.7)	16.7 (5.9)	2.44^*^
**SEXUAL RELATIONSHIP STATUS**^a^			
Exclusive	50.0	30.4	2.06
Non-exclusive	13.6	56.5	
Not sexually active	36.4	13.1	
Number of sexual partners^b^	2.5 (3.5)	20.9 (27.5)	3.11^**^
Frequency of sexual intercourse per week^b^	0.5 (0.7)	3.7 (2.6)	5.58^***^
Frequency of masturbation per week^b^	1.7 (0.9)	5.1 (3.2)	4.80^***^
Frequency of viewing pornography per week^b^	2.3 (0.6)	5.5 (2.7)	5.42^***^
SAST–R^b^	0.5 (0.9)	11.3 (3.3)	14.82^***^
HBI^b^	26.9 (13.5)	54.4 (7.3)	8.55^***^

*PHB, Problematic Hypersexual Behavior; WAIS-R, Wechsler Adult Intelligence Scale-Revised; SAST-R, Sexual Addiction Screening Test revised; HBI, Hypersexual Behavior Inventory*.

a *Data are presented as percentages of the total cohort and analyzed using a chi-square test*.

b *Data are represented as means with standard deviations and analyzed using an independent sample t-test. Data reflect information regarding behaviors in the previous 6 months*.

* *p < 0.05*,

** p < 0.01, and

*** *p < 0.001*.

### Task and experimental paradigm

**Figure 1:**
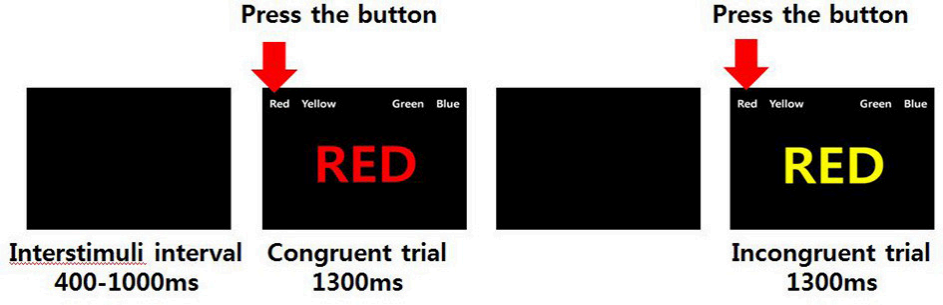
Examples of congruent and incongruent conditions in the Stroop task.

The Stroop test is named after John Ridley Stroop (31), who is credited with the first English publication of the effects associated with incongruent stimuli. The present study used a modified version of the Stroop task developed by Peterson et al. (32) during fMRI scanning. Participants held one of two keypads, each equipped with two response buttons, in each hand. We tried to eliminate any effects (e.g., effect of handedness, Simon effect) that was induced during the experiment. To eliminate the effects, we had 24 different stimuli per one word that show the location of the color button on the keypad. The one example out of 24 stimuli is the Figure 1 as the order of color button was Red, Yellow, Green, Blue. During the experiment, the order of color button was randomly presented out of 24 stimuli per each trial. By repeating the task, we were also able to collect more data to increase the reliability of the results. Participants practiced one run before the scanning session, and they all indicated that they had a clear understanding of the task. Stimuli were presented via an overhead mirror during fMRI scanning.

The Stroop task was divided into congruent and incongruent conditions. In the congruent condition, a word in a semantically matched color (e.g., the word “RED” in red color) was displayed on a screen, and participants were instructed to press the corresponding color button as quickly as possible. In the incongruent condition, a word with unmatched meaning and color (e.g., the word “RED” in yellow color) was displayed on the screen, and participants were instructed to press the color button that corresponded to the color of the word while ignoring the meaning of the word. The target stimulus was presented in the center of the display screen. Four possible answers (color words in white font) were presented above it (in the upper visual field) to minimize contextual memory demands, as shown in Figure 1.

The order of events and time for each condition were as follows: (1) first, an instruction alerting the participant to the start of the experiment was presented for 6 s; (2) second, an empty black screen was presented for a random interval of 400–1,000 ms as the inter-stimulus interval; (3) third, a stimulus (congruent trial or incongruent trial) was presented for 1,300 ms; and (4) lastly, an empty screen was presented again for 4,000 ms.

The Stroop task of the present study was designed as an event-related paradigm and comprised 130 congruent conditions plus 85 incongruent conditions presented in a randomized order. The task was repeated twice, and each task lasted 444 s. Examples of the Stroop stimuli and the fMRI paradigm are shown in Figure 1.

### Imaging acquisition

An echo-planar imaging blood oxygen level-dependent (EPI-BOLD) method was used to acquire brain images. The parameters for image acquisition were as follows: repetition time/echo time = 2,000/28 ms; field of view = 240 × 240 mm; matrix size = 64 × 64; slice thickness = 5 mm, no gap; and flip angle = 80°. The overall volume of each experimental session was 222 images, and included three dummy images acquired across 6 s. T1-weighted images were collected as structural images with the following acquisition parameters: repetition time/echo time = 280/14 ms; FOV = 240 × 240 mm, matrix size = 256 × 256; slice thickness = 4 mm; and flip angle = 60°. The imaging plane was positioned parallel to the anterior commissure-posterior commissure line.

### Statistical analyses

#### Behavioral data analysis

The mean response times and percentages of correct responses were calculated in each condition. To normalize the distribution of response time data, we transformed the response time using the following equation: log (1/response time) (33). The log-transformed response time was used for the two-way analysis of variance (ANOVA) with group as the between-subjects factor (i.e., participants with PHB vs. healthy controls) and condition as the within-subjects factor (i.e., congruent vs. incongruent stimuli).

The percentages of correct responses (i.e., hit rates) between conditions in each group and between groups in each condition were analyzed non-parametrically using the Wilcoxon rank sum test or Mann-Whitney U test (*p* < 0.05). All analyses were conducted using SPSS version 20.0 (IBM Corp., Armonk, NY, USA).

#### Imaging data analysis

Statistical Parametric Mapping version 8 (SPM 8, Wellcome Department of Imaging Neuroscience, London, UK) was used to analyze brain imaging data. Functional data were realigned to the first scan of each session as a reference using three-dimensional rigid body registration with six degrees of freedom. Then, the realigned scans were coregistered to each participant's anatomical image and normalized to the MNI (Montreal Neurologic Institute) coordinate system. To decrease spatial noise, data were smoothed using an 8-mm isotropic Gaussian kernel.

After preprocessing, a design matrix was constructed for each condition in each participant. When constructing the design matrix, degrees of head movement/rotation during head movement compensation were added as regression variables to increase the signal-to-noise ratio. Then, z-maps were generated according to the stimulus condition (congruent and incongruent) for each individual. To identify specific brain regions exhibiting different patterns of activity between individuals with PHB and healthy controls, an ANOVA was conducted using condition (congruent vs. incongruent) as the within-group variable and group (individuals with PHB vs. controls) as the between-group variable [false discovery rate (FDR)-corrected, *p* < 0.05].

Based on previous neuroimaging studies on Stroop task and addicts and the results of the ANOVA, the dorsolateral prefrontal cortex (DLPFC) and inferior parietal cortex were selected as regions of interest (ROIs) (21–25).

To extract percent signal changes from ROIs, the MarsBaR 0.42 program (http://www.sourceforge.net/projects/marsbar) was used in an SPM toolbox (http://www.fil.ion.ucl.ac.uk/spm/ext). The ROIs were defined by centering spheres on the respective peak voxels with a radius of 5 mm for all activated areas in the interaction results (FDR-corrected, *p* < 0.05). To compare these values between groups with follow-up *t*-tests, the percent signal change was extracted for each subject, and a two-way ANOVA was performed using SPSS version 20. To evaluate the relationship between PHB severity and neural responses to the Stroop interference, correlation analyses were performed between percent signal changes from the ROIs during the incongruent condition and the scores of standardized measurements (i.e., SAST-R and HBI scores).

## Results

### Behavioral results

**Figure 2:**
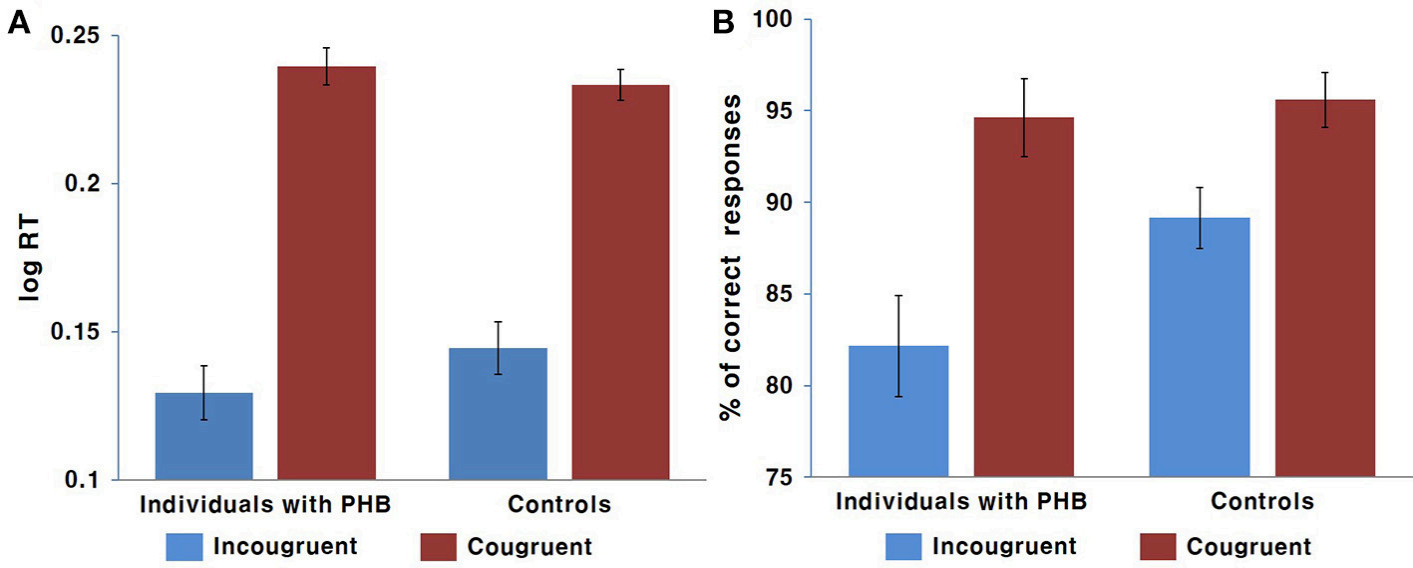
Behavioral results. **(A)** Mean response time in ms. **(B)** Mean response accuracy as a percentage. Error bars indicate the standard error of the mean.

A two-way ANOVA revealed a significant main effect of condition [*F*_(1, 43)_ = 171.43, *p* < 0.001, Cohen's *f* = 3.99], indicating that the response was generally slower in the incongruent condition compared to that in the congruent condition. There was no significant interaction effect between condition and group [*F*_(1, 43)_ = 0.34] or main effect of group [*F*_(1, 43)_ = 1.98, Figure 2].

The non-parametric Wilcoxon test indicated a significant accuracy difference between the congruent and incongruent conditions in both the PHB (*Z* = −6.39, *p* < 0.05) and control (*Z* = 5.71, *p* < 0.05) groups, indicating that there was generally a higher incidence of error responses in the incongruent condition. We also identified significant differences in performance accuracy between groups for the incongruent condition (*Z* = −2.12, *p* < 0.05), indicating that the healthy controls performed better than the PHB group; however, there were no significant between-group differences in response accuracy for the congruent condition (*Z* = −1.48, Figure 2). These data indicate that both groups responded accurately to congruent conditions, whereas participants with PHB were more likely to respond inaccurately in conditions that required inappropriate incongruent effects to be ignored.

### Imaging results

#### Main effect of condition

A main effect of condition (congruent vs. incongruent) was observed in the right putamen, right middle frontal gyrus, and right inferior frontal gyrus (*p* < 0.05, FDR-corrected; Table **3**). These regions exhibited greater activation under incongruent than under congruent conditions. However, no brain regions were activated more by the congruent than by the incongruent condition.

#### Main effect of group

A main effect of group (PHB group vs. controls; *p* < 0.05, FDR-corrected; Table 2) was observed in the bilateral inferior parietal areas, right middle frontal gyrus, and right inferior frontal gyrus. The control group exhibited increased activation in the bilateral inferior parietal areas and the right middle and inferior frontal gyri relative to the PHB group (*p* < 0.05, FDR-corrected; Table 3). No brain regions were activated more in the PHB group than in controls.

**Table 2:** Mean hit rates and response latencies in Stroop test conditions.

	**Control group**	**PHB group**
	**(*n* = 22)**	**(*n* = 23)**
Hit rate in congruent conditions	97.35 (3.29)	95.74 (8.99)
Hit rate in incongruent conditions	89.45 (14.37)	82.14 (22.01)
Response time in congruent conditions	601.77 (81.44)	602.04 (61.44)
Response time in incongruent conditions	762.00 (140.14)	784.35 (126.27)

**Table 3:** Imaging results: main effects of condition and group (*p* < 0.05, FDR-corrected).

**Brain regions**	**Side**	**No. of voxels in cluster**	***F***	**x, y, z MNI coordinates**		
**MAIN EFFECT OF CONDITION**						
**Incongruent** > **Congruent**						
Putamen	R	63	2.61	30	−8	−6
Middle/inferior frontal gyrus (BA 8, 9, 47)	R	51	3.07	52	14	30
		33	2.96	52	21	2
**Congruent** > **Incongruent**						
No regions						
**MAIN EFFECT OF GROUP**						
**Control Group** > **PHB Group**						
Inferior parietal cortex (BA 40)	R, L	85	4.01	42	−37	38
				−42	−36	48
Middle/inferior frontal gyrus	R	47	2.54	42	25	38
(BA 9)						
**PHB Group** > **Control Group**						
No regions						

*PHB, problematic hypersexual behavior; R, right; L, left; BA, Brodmann area*.

#### Condition × group interaction effects

**Figure 3:**
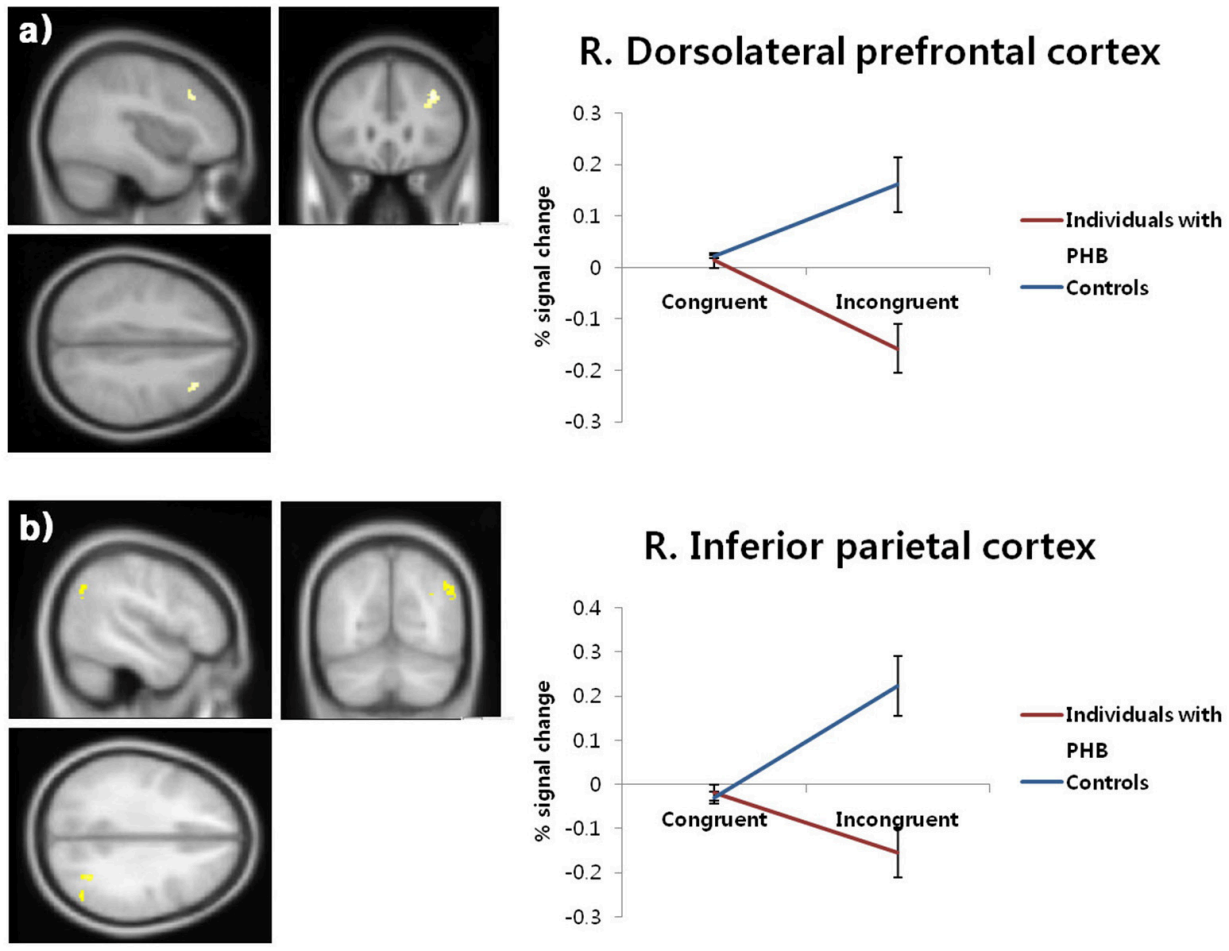
Brain activation patterns in the right dorsolateral prefrontal cortex **(a)** and right inferior parietal cortex **(b)**. The graphs depict the extracted signal change averaged across voxels from each region, displaying condition × group interactions (*p* < 0.05, FDR-corrected). FDR, false discovery rate; PHB, problematic hypersexual behavior; R. DLPFC, right dorsolateral prefrontal cortex; R. IPC, right inferior parietal cortex.

Significant condition × group interactions (*p* < 0.05, FDR-corrected; Table 4, Figure 3) were identified in the right DLPFC and right inferior parietal cortex.

**Table 4:** Imaging results: interaction effects of option × group (*p* < 0.05, FDR-corrected).

**Brain ROIs**	**Side**	**No. of voxels in cluster**	***F***	**x, y, z MNI**		
				**coordinates**		
**INCONGRUENT CONDITIONS**						
**Control Group** > **PHB Group**						
Dorsolateral prefrontal cortex	R	35	2.28	40	28	38
Inferior parietal cortex	R	66	2.35	48	−66	32

*PHB, problematic hypersexual behavior; ROI, region of interest*.

In follow-up *t*-tests using the extracted BOLD signal changes for each ROI, participants with PHB exhibited significantly less activation in the right DLPFC in the incongruent condition [*t*_(43)_ = 4.46, *p* < 0.01, Cohen's *d* = 1.33] relative to healthy controls, whereas no significant group difference was found in the congruent condition [*t*_(43)_ = 0.48, *p* > 0.05, Cohen's *d* = 0.14; Figure 3a]. A similar pattern of brain activation was observed in the right inferior parietal cortex: Compared to controls, individuals with PHB exhibited diminished activation in the right inferior parietal cortex during the incongruent conditions [*t*_(43)_ = 4.28, *p* < 0.01, Cohen's *d* = 1.28], but no significant group difference was observed during the congruent conditions [*t*_(43)_ = 0.60, *p* > 0.05, Cohen's *d* = 0.18; Figure 3b].

#### Correlation analyses

To confirm the functions of ROIs in cognitive control, we conducted the correlation analyses between the behavioral data (i.e., response time and response accuracy) and BOLD signal changes for each ROI (i.e., the right DLPFC and right inferior parietal cortex). There are significant correlations between them (Supplementary Materials, Figure S1).

**Figure 4:**
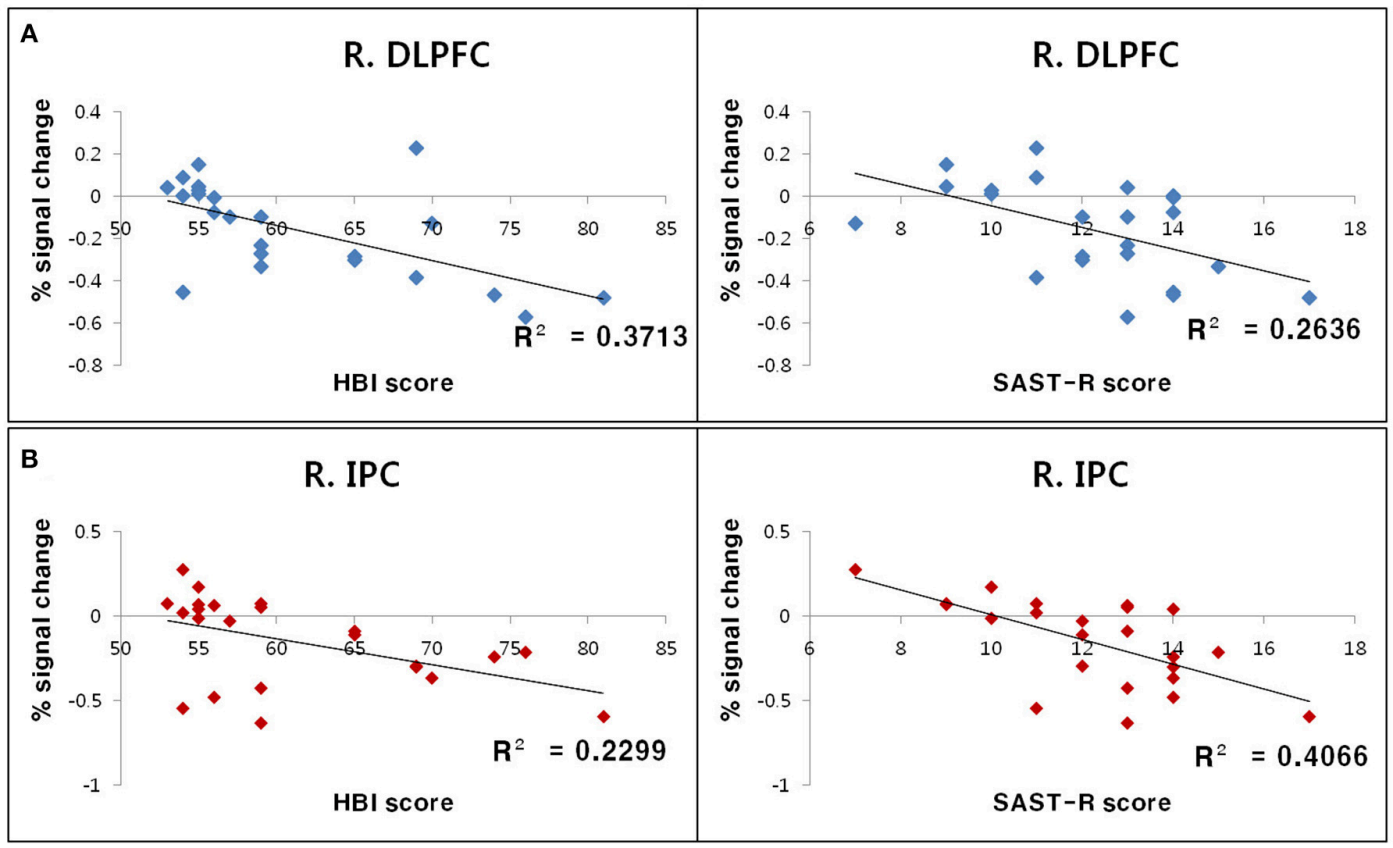
Results of correlation analyses between standardized measurement scores and BOLD signal changes in ROIs during the incongruent Stroop condition. **(A)** Negative correlations between the percent signal change in the R. DLPFC and HBI score (left) as well as SAST-R score (right). **(B)** Negative correlations between the percent signal change in the R. IPC right and HBI score (left), as well as SAST-R score (right). BOLD, blood oxygen level-dependent; HBI, Hypersexual Behavior Inventory; R. DLPFC, right dorsolateral prefrontal cortex; R. IPC, right inferior parietal cortex; ROI, region of interest; SAST-R, Sexual Addiction Screening Test-R.

The relationship between standardized measurement scores (i.e., SAST-R and HBI scores) and BOLD signal changes for each ROI (i.e., the right DLPFC and right inferior parietal cortex) were calculated for all participants with PHB. Negative correlations were observed between standardized measurement scores and BOLD signal changes in the right inferior parietal cortex (SAST-R: *r* = −0.64, *n* = 23, *p* < 0.01; HBI: *r* = −0.48, *n* = 23, *p* < 0.01) and right DLPFC (SAST-R: *r* = −0.51, *n* = 23, *p* < 0.01; HBI: *r* = −0.61, *n* = 23, *p* < 0.01; Figure 4).

## Discussion

The present study aimed to elucidate the neural mechanisms underlying impairments in executive control among individuals with PHB. As hypothesized, individuals with PHB exhibited diminished executive control associated with decreased activation of the DLPFC and right inferior parietal cortex during incongruent Stroop trials. Furthermore, decreased BOLD signal changes in the DLPFC and inferior parietal cortex during incongruent Stroop trials were associated with higher SAST-R and HBI scores in individuals with PHB. We also identified other brain areas besides the region of interest (DLPFC) during the Stroop task. The right putamen in the basal ganglia and middle and inferior frontal gyri were more activated during the incongruent condition compared to the congruent condition, which is consistent with previous studies of the Stroop effect (32, 34). The group differences in the inferior parietal cortex and middle and inferior frontal gyri during the Stroop task are in line with results from patients with other addictive behaviors (35).

With regard to task performance, individuals with PHB exhibited higher error rates than healthy controls in the incongruent condition. The Stroop task requires the cognitive inhibition of automatic responses (e.g., word reading); specifically, the target action in the incongruent condition can only be performed correctly if the incongruent stimulus (the word's meaning) is cognitively inhibited. Shorter response times and increased response accuracy are thought to reflect better cognitive flexibility and inhibition (36). Therefore, poor performance in individuals with PHB can be interpreted as reflecting impaired executive control. This observation is consistent with the findings of previous studies regarding behavioral addiction (15, 16).

Based on the results of this study, we infer that the behavioral characteristics of PHB may be due to decreased activity in the right DLPFC and right inferior parietal cortex. Goldstein and Volkow (25) suggested that slower task performance and higher error rates during incongruent Stroop task conditions are a hallmark of PFC dysfunction. Studies evaluating the Stroop task in addiction (i.e., substance dependence and behavioral addiction) have reported reduced activity in the right PFC, including the DLPFC, during incongruent conditions compared to congruent conditions (15, 26, 37, 38). The findings of the current study are consistent with these previous reports and further elaborate upon their results by showing a negative correlation between activation of these brain areas and PHB severity.

The DLPFC is associated with higher-order cognitive control functions such as monitoring and manipulating information in working memory (39). Milham et al. (40) proposed two roles for the DLPFC during Stroop task performance: (1) biasing the selection of task-relevant representations within working memory, and (2) modulating activity in a posterior processing system (e.g., amplifying neural activity within the task-relevant processing system). The former role refers to the process of discriminating, selecting, and manipulating task-relevant (i.e., graphic) rather than task-irrelevant (i.e., semantic) information. The latter role describes the process of activating brain regions in the task-relevant processing system in order to allocate and maintain attentional resources for the discrimination of task-relevant information. The DLPFC is closely interconnected with the posterior visual processing area (e.g., the parietal lobe and primary visual cortex) and is thought to amplify neural activity via these direct neuronal connections (41–44). Brain imaging studies revealed that DLPFC activation is accompanied by activation of the parietal lobe during incongruent Stroop conditions (21, 22, 45). These data are supported by the results of the present study, which identified co-activation of the DLPFC and parietal lobe in the control group during incongruent conditions. The inferior parietal cortex is associated with visual attention (46) and helps to maintain selective attentional control by allowing one to disregard irrelevant stimuli. In one study of working memory task performance, increasing levels of incongruent stimuli produced greater activation of the posterior parietal cortex (47). Therefore, reduced activity in the right DLPFC and inferior parietal cortex in individuals with PHB might represent deficits in the ability to discriminate relevant information and disregard irrelevant information. These deficits in executive control may make it more difficult for individuals with PHB to suppress sexual cravings or behaviors.

The limitations of the present study are as follows. First, this study only evaluated the current mental status of individuals with PHB; therefore, our results do not address the causal nature of the relationship between executive control deficits and PHB. Second, we used the SAST and HBI scales to evaluate participant hypersexuality. They measure constructs related to psychological factors such as sexual motivation and sexual shame, as well as those related with sexual behavioral factors including frequency. Recent studies on sex and pornography addiction suggest that psychological factors are more important than sexual behavioral factors to develop addictive behaviors (48–50). These findings indicate a possibility for different effects between psychological factors and behavioral factors in executive control of sex and pornography addiction. Therefore, it is important to determine how each factor affects executive control and identify which are more important in developing the sex and pornography addiction. In future studies, we plan to test the associations between each factor and executive control by eliminating the confounding effects of other factors. Third, this study only investigated heterosexual Asian male participants. Future studies should include participants of different genders, sexual orientations, and ethnic backgrounds to provide more generalizable insights into PHB. Although the individuals with PHB in this study met the proposed criteria for PHB used in previous studies (2, 28), there are no formal diagnostic criteria for PHB. Thus, a clinical diagnostic definition of PHB is required in order to improve the reliability of PHB studies. Finally, it would be interesting to identify whether the findings are the same for the PHB group with thoughts (e.g., fantasies) only vs. individuals who actually engage in problematic behaviors. However, the sample size in this study was relatively small, and our participants had a high level of sexual fantasies and also frequently engaged in problematic behaviors. For that reason, it was hard to distinguish the two groups. We hope to include this group comparison in future studies by recruiting more subjects.

Despite the aforementioned limitations, the present study is useful for understanding the characteristics and relevant neural mechanisms of PHB. In summary, individuals with PHB show poorer task performance and decreased activation in the PFC during the Stroop interference task compared to normal controls. Our findings validate the presence of impaired executive control and possible prefrontal dysfunction in individuals with PHB, similar to findings in other problematic excessive behavior conditions.

## Ethics statement

All participants provided their written informed consent after being thoroughly informed about the details of the experiment. The Chungnam National University Institutional Review Board (IRB) approved the experimental and consent procedures (approval number: 01309-SB-003-01; Daejeon, South Korea). All participants received financial compensation (50 US dollars) for their participation.

## Author contributions

J-WS contributed to conception and experimental design, or acquisition of data, or analysis, and interpretation of data, and J-HS contribute substantially to interpretation of data and drafted the article or revised it critically for important intellectual content.

### Conflict of interest statement

The authors declare that the research was conducted in the absence of any commercial or financial relationships that could be construed as a potential conflict of interest.

## Abbreviations

DLPFC: dorsolateral prefrontal cortex
EPI_BOLD: echo-planar imaging blood oxygen level-dependent
HBI: Hypersexual Behavior Inventory
PHB: problematic hypersexual behavior
SAST: Sexual Addiction Screening Test.
