# Supplementary material for: Altered Prefrontal and Inferior Parietal Activity During a Stroop Task in Individuals With Problematic Hypersexual Behavior
Source: Front Psychiatry. 2018 Sep 25;9:460. doi: 10.3389/fpsyt.2018.00460 (PMC6167473; doi:10.3389/fpsyt.2018.00460)
Supplement: Supplementary file 1 [file Data_Sheet_1.docx]

Supplementary Materials

Altered Prefrontal and Inferior Parietal Activity During a Stroop Task in Individuals with Problematic Hypersexual Behavior

Ji-woo Seok, Jin-Hun Sohn*

*** Correspondence:** Jin-Hun Sohn: jhsohn@cnu.ac.kr

# Supplementary Data

# Participants

# Potential PHB subjects were recruited from treatment facilities for problematic sexual behavior and Sex Addiction Anonymous meetings. Clinical interviews of all potential PHB subjects were conducted to identify those who fulfilled the inclusion criteria and did not meet the exclusion criteria. We had an interview to confirm the pure effect of hypersexuality on the Stroop task with several exclusion criteria based on previous study (Anand et al., 2005; Chen et al., 2005; LeMarquand et al., 1998; Kaufman et al., 2003; Hester and Garavan, 2004). Anyone who met the followings criteria was excluded (1) who is over 45 years old or under 18 years old; (2) has other psychiatric disorders based on DSM-5 within the past 12 months (e.g., alcohol use disorder, gambling disorder, major depressive disorder, bipolar disorder, obsessive-compulsive disorder, etc); (3) is a current user of medication which might cause a pharmacological effect in the brain vascular system and has a history of serious head injury; (d) is a homosexual male; (e) has criminal records proven to be impulsive and has problems in the response inhibition (Chen et al., 2005; LeMarquand et al., 1998), and (f) is ineligible for MRI scan (i.e., has metal in the body, severe astigmatism, claustrophobia). As a result, 23 men with the PHB (mean age = 26.12, SD = 4.11) were recruited with the above criteria. Finally, 23 male participants were selected; 21 were heterosexual and 2 were bisexual.

# 2 Supplementary Tables

Table S1. PHB diagnostic criteria

|  | Diagnostic criteria |
| --- | --- |
| Sexual addiction (Carnes et al., 2010) | Three or more of the following symptoms:   1. Recurrent failure to resist impulses to engage in specific sexual behavior 2. Frequent engaging in sexual behaviors to a greater extent or over a longer period of time than intended 3. Persistent desire or unsuccessful efforts to stop, reduce, or control sexual behaviors 4. Inordinate amount of time spent in obtaining sex, being sexual, or recovering from sexual experience 5. Preoccupation with sexual behavior or preparatory activities 6. Frequent engaging in sexual behavior when expected to fulfill occupational, academic, domestic, or social obligations 7. Continuation of sexual behavior despite knowledge of having a persistent or recurrent social, financial, psychological, or physical problem that is caused or exacerbated by the behavior 8. Need to increase the intensity, frequency, number, or risk of sexual behaviors to achieve the desired effect, or diminished effect with continued sexual behaviors at the same level of intensity, frequency, number, or risk 9. Giving up or limiting social, occupational, or recreational activities because of sexual behavior 10. Distress, anxiety, restlessness, or irritability if unable to engage in sexual behavior |
| A proposed criteria of hypersexual disorder for DSM-V (Kafka, 2010) | A. Over a period of at least 6 months, recurrent and intense sexual fantasies, sexual urges, or sexual behaviors in association with 3 or more of the following 5 criteria:   - A1. Time consumed by sexual fantasies, urges, or behaviors repetitively interferes with other important (non-sexual) goals, activities, and obligations   A2. Repetitively engaging in sexual fantasies,  urges or behaviors in response to dysphoric  mood states (e.g., anxiety, depression,  boredom, irritability)  A3. Repetitively engaging in sexual fantasies, urges or behaviors in response to stressful life events  A4. Repetitive but unsuccessful efforts to control or significantly reduce these sexual fantasies, urges, or behaviors  A5. Repetitively engaging in sexual behaviors while disregarding the risk for physical or emotional harm to self or others  B. There is clinically significant personal distress or impairment in social, occupational, or other important areas of functioning associated with the frequency and intensity of these sexual fantasies, urges, or behaviors  C. These sexual fantasies, urges, or behaviors are not due to the direct physiological effect of an exogenous substance (e.g., a drug of abuse  or a medication)  Specify if: masturbation, pornography, sexual behavior with consenting adults, cybersex, telephone sex, strip clubs |

# 3. Supplementary Result

*Correlation analyses.* To confirm the functions of ROIs in cognitive control, we conducted the correlation analyses between the behavioral data (i.e., response time and response accuracy) and BOLD signal changes for each ROI (i.e., the right dorsolateral prefrontal cortex and right inferior parietal cortex). Positive correlations were shown between behavioral data and BOLD signal changes in the right inferior parietal cortex (response time: r = 0.40, n = 45, *p* < 0.01; HBI: r = 0.48, n = 45, *p* < 0.001) and right dorsolateral prefrontal cortex (response time: r = 0.48, n = 45, *p* < 0.01; HBI: r = 0.60, n = 45, *p* < 0.001)

# 4. Supplementary Figure


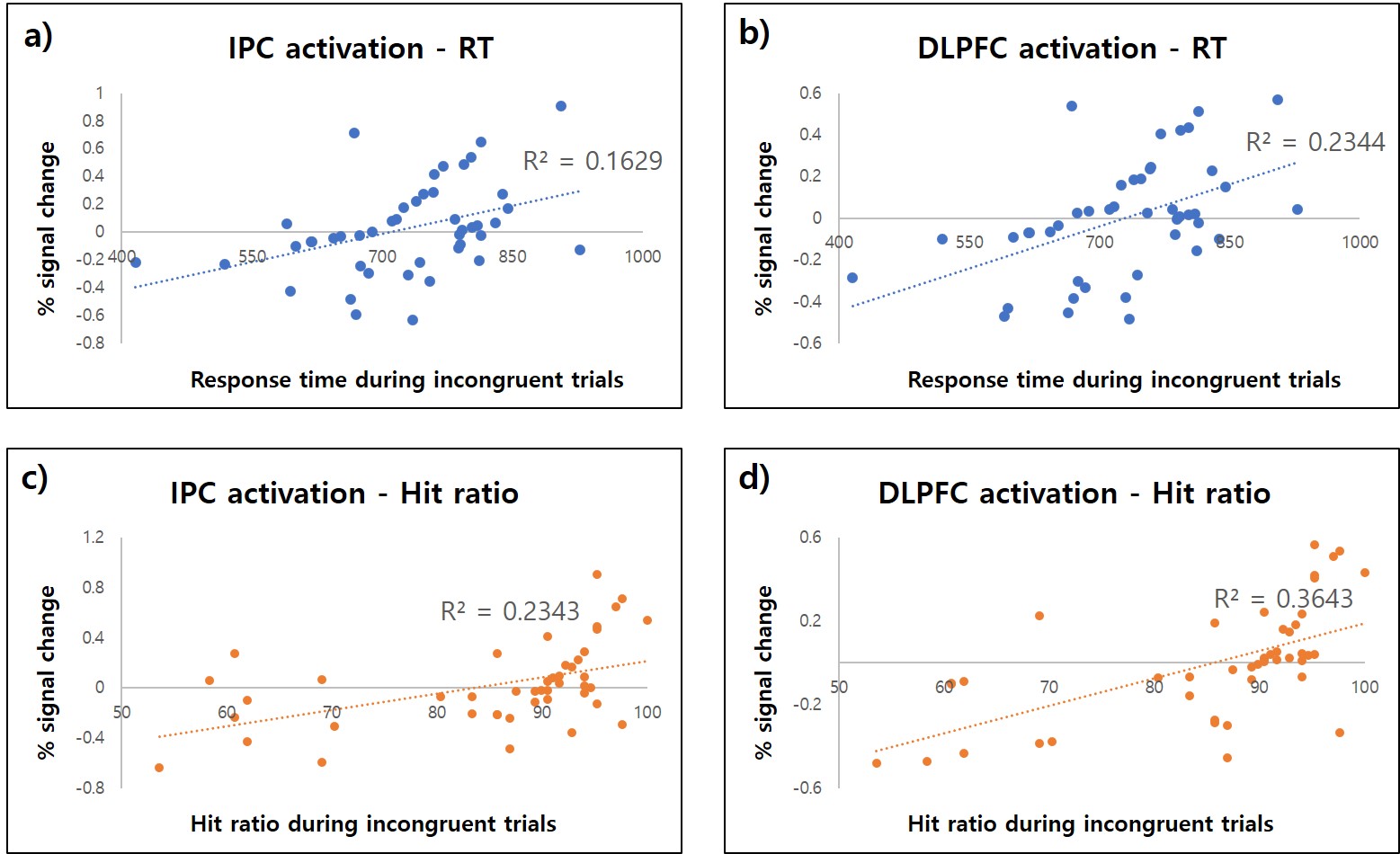


Figure S1. Results of correlation analyses between behavioral data and BOLD signal changes in ROIs during the incongruent Stroop condition. (a) Positive correlations between the percent signal change in the R. IPC and response time. (b) Positive correlations between the percent signal change in the R. DLPFC and response time. (c) Positive correlations between the percent signal change in the R. IPC and response accuracy. (d) Positive correlations between the percent signal change in the R. DLPFC and response accuracy. Abbreviations: BOLD, blood oxygen level-dependent; R. DLPFC, right dorsolateral prefrontal cortex; R. IPC, right inferior parietal cortex; ROI, region of interest; RT, response time.

# 4. Supplementary References

# Anand, A., Li, Y., Wang, Y., Wu, J., Gao, S., Bukhari, L., Mathews, V.P., Kalnin, A., Lowe, M.J., 2005. Antidepressant effect on connectivity of the mood-regulating circuit: an FMRI study. Neuropsychopharmacology. 30, 1334–1344. DOI: https://doi.org/10.1038/sj.npp.1300725

# Chen, C.-Y., Tien, Y.-M., Juan, C.-H., Tzeng, O.J., Hung, D.L. 2005. Neural correlates of impulsive-violent behavior: an event-related potential study. Neuroreport. 16, 1213–1216.

# LeMarquand, D.G., Pihl, R.O., Young, S.N., Tremblay, R.E., Séguin, J.R., Palmour, R M., Benkelfat, C., 1998. Tryptophan depletion, executive functions, and disinhibition in aggressive, adolescent males. Neuropsychopharmacology. 19, 333–341. DOI: https://doi.org/10.1016/S0893-133X(98)00029-3

# Kaufman, J.N., Ross, T.J., Stein, E.A., Garavan, H., 2003. Cingulate hypoactivity in cocaine users during a GO-NOGO task as revealed by event-related functional magnetic resonance imaging. J. Neurosci. 23, 7839–7843.

# Hester, R., Garavan, H., 2004. Executive dysfunction in cocaine addiction: evidence for discordant frontal, cingulate, and cerebellar activity. J. Neurosci. 24, 11017–11022. DOI: https://doi.org/10.1523/JNEUROSCI.3321-04.2004
